# Supplementary material for: β-Arrestin 1 Differentially Modulates cAMP and ERK Pathways Downstream of the FSH Receptor
Source: Curr Issues Mol Biol. 2025 Dec 16;47(12):1051. doi: 10.3390/cimb47121051 (PMC12732243; doi:10.3390/cimb47121051)
Supplement: Supplementary file 1 [file cimb-47-01051-s001.zip › cimb-4009859-supplementary.pdf]

Supplementary Data

Supplementary Table S1 Monkey FSH single chain primer sequences

DNA	Primer Name	Primer sequence
FSH β	Cyn_a1F-EcoRI	5’-GAATTCGCCACCATGGATTACTACAGAAAATATGCA -3’
	CynFSH b R	5’-TCCATCAGGAAATTCTTTTTCACTGAAGGA -3’
FSH α	Cyn_a1 F	5’-TTTCCTGATGGAGAGTTTACAATGCAGGAT -3’
	a2-5 R	5’-GCGGTCGACTTAAAATTTGTGATAATAACA -3’

Supplementary Table S2 Primer sequences

Primer Name	Primer Sequence
MonkeyFSH-GGSA-HIS R1	5’-GTGAGCACTACCGCCAAATTTGTGATAATA-3’
MonkeyFSH-GGSA-HIS R2	5’-GTGATGGTGATGGTGGTGAGCACTACCGCC-3’
MonkeyFSH-GGSA-HIS R3	5’-CTCGAGTCAGTGATGGTGATGGTGATGGTG-3’
MonkeyFSH-GGSA-strep R1	5’-CCAAGCACTACCGCCAAATTTGTGATAATA-3’
MonkeyFSH-GGSA-strep R2	5’-GAACTGCGGGTGGCTCCAAGCACTACCGCC-3’
MonkeyFSH-GGSA-strep R3	5’-CTCGAGTCATTTTTCGAACTGCGGGTGGCT-3’
